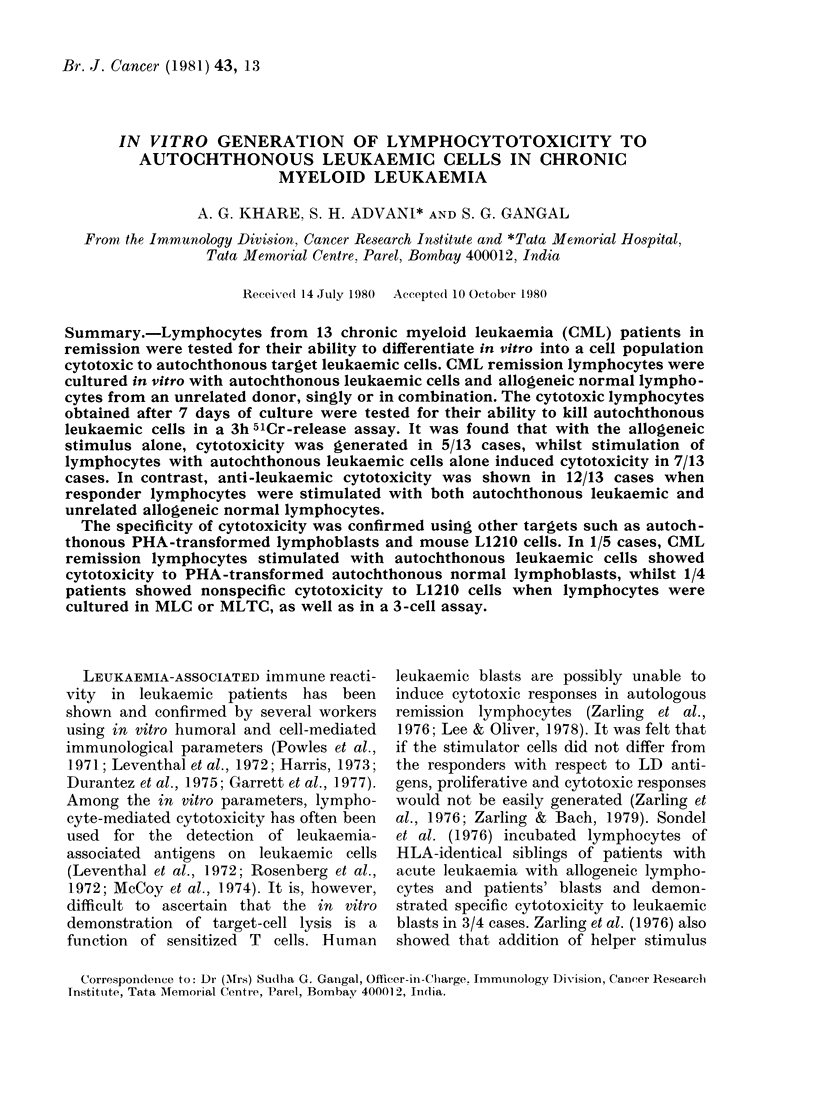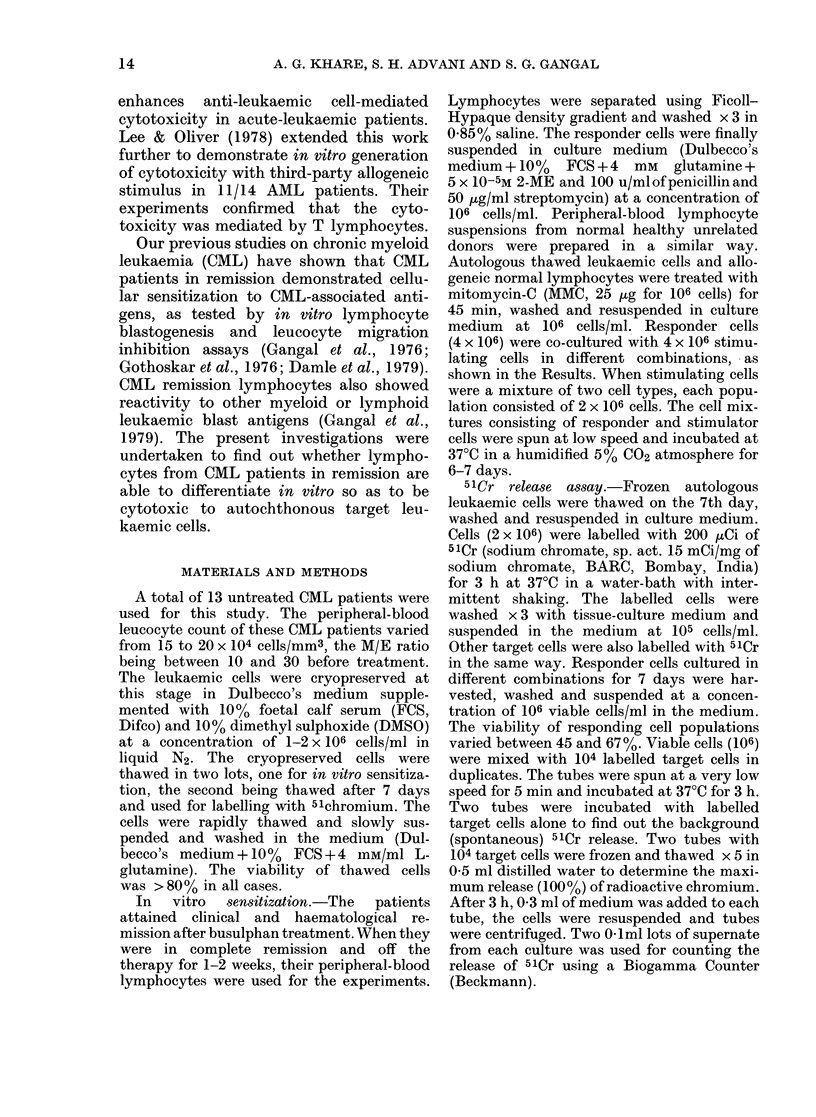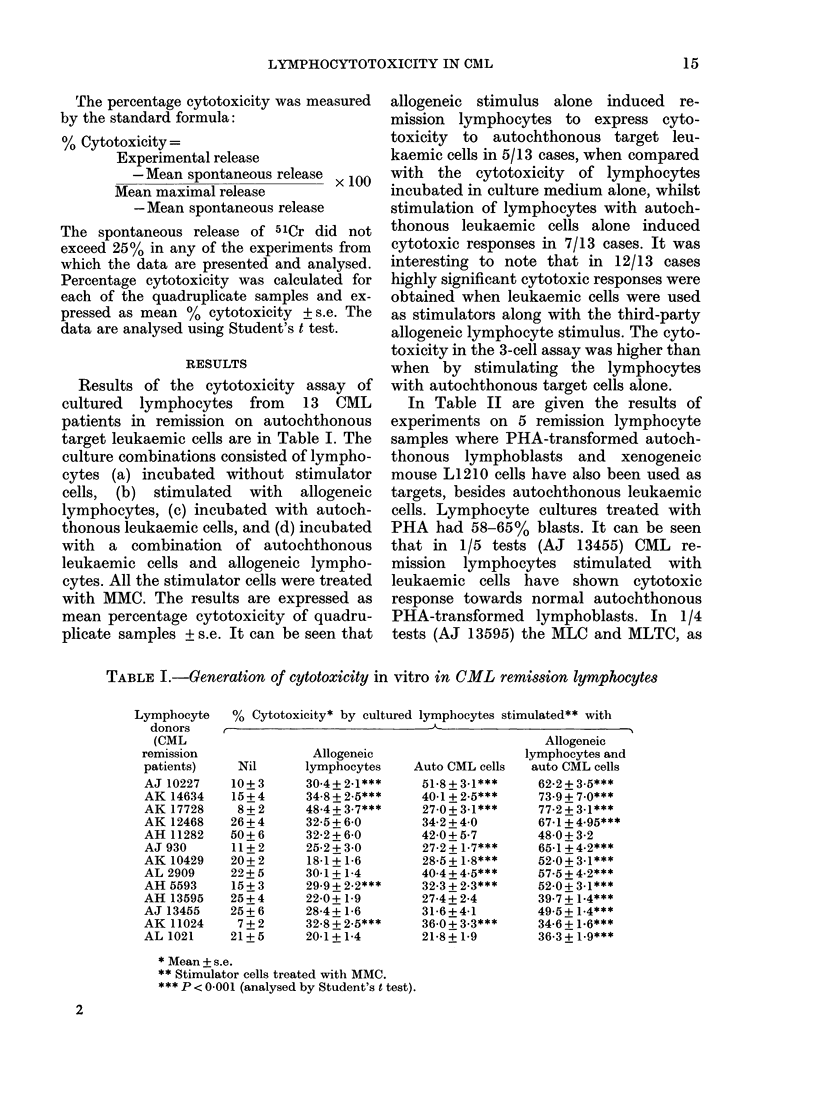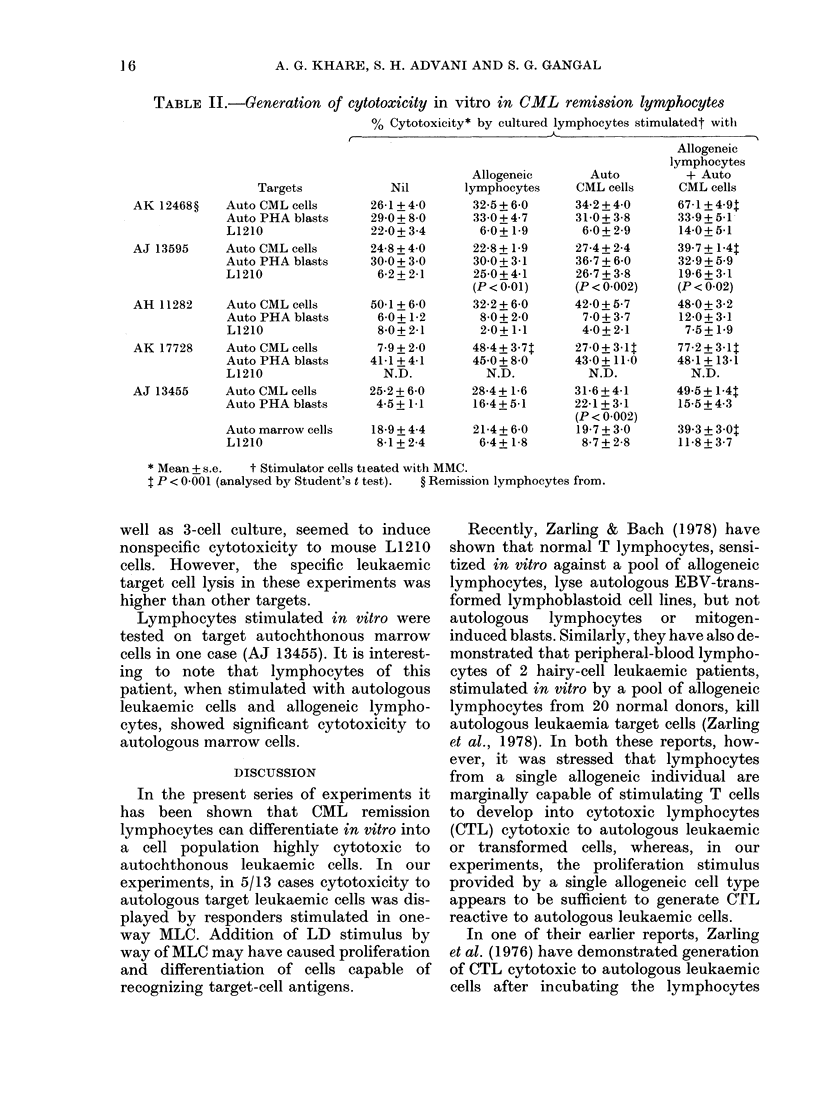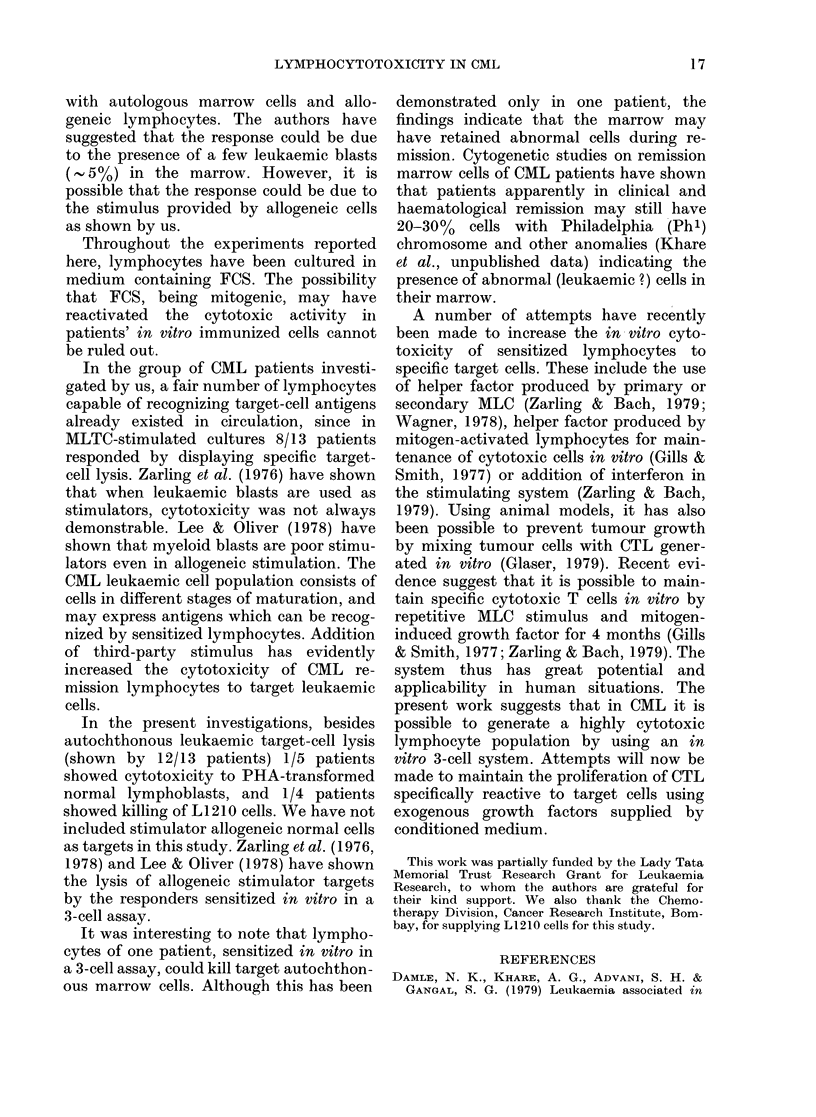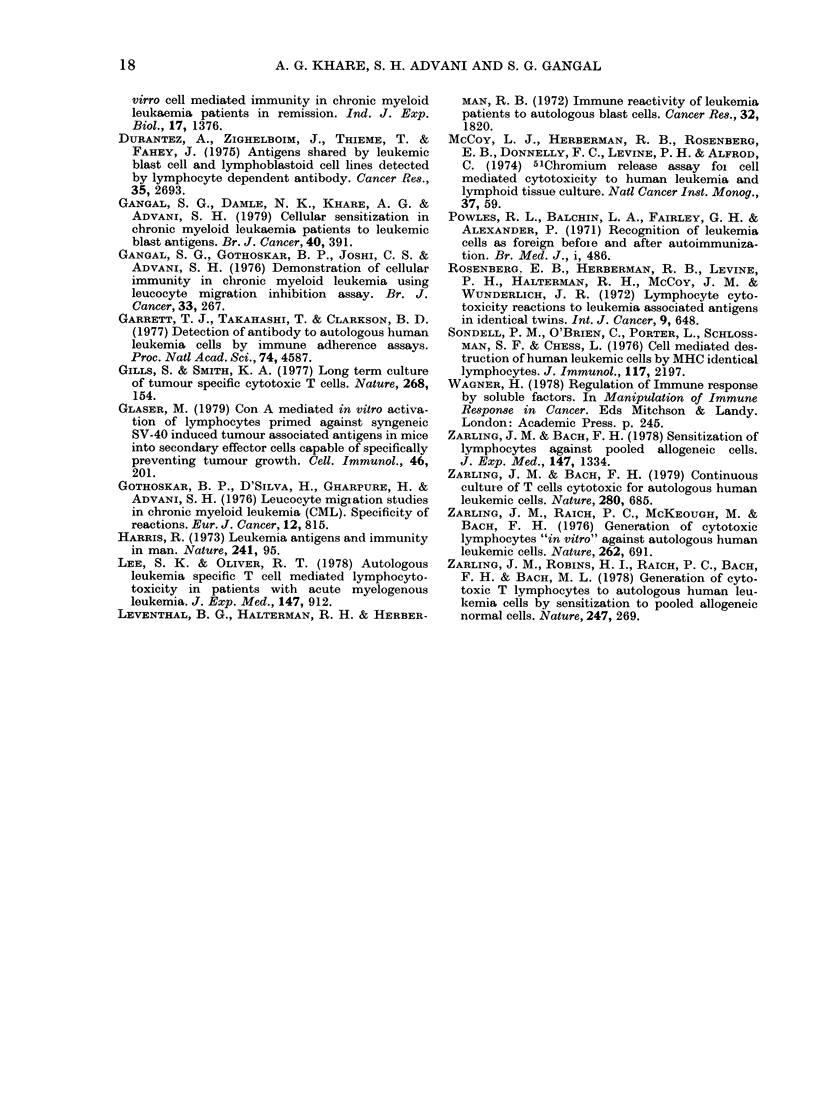# In vitro generation of lymphocytotoxicity to autochthonous leukaemic cells in chronic myeloid leukaemia.

**DOI:** 10.1038/bjc.1981.3

**Published:** 1981-01

**Authors:** A. G. Khare, S. H. Advani, S. G. Gangal

## Abstract

Lymphocytes from 13 chronic myeloid leukaemia (CML) patients in remission were tested for their ability to differentiate in vitro into a cell population cytotoxic to autochthonous target leukaemic cells. CML remission lymphocytes were cultured in vitro with autochthonous leukaemic cells and allogeneic normal lymphocytes from an unrelated donor, singly or in combination. The cytotoxic lymphocytes obtained after 7 days of culture were tested for their ability to kill autochthonous leukaemic cells in a 3h 51Cr-release assay. It was found that with the allogeneic stimulus alone, cytotoxicity was generated in 5/13 cases, whilst stimulation of lymphocytes with autochthonous leukaemic cells alone induced cytotoxicity in 7/13 cases. In contrast, anti-leukaemic cytotoxicity was shown in 12/13 cases when responder lymphocytes were stimulated with both autochthonous leukaemic and unrelated allogeneic normal lymphocytes. The specificity of cytotoxicity was confirmed using other targets such as autochthonous PHA-transformed lymphoblasts and mouse L1210 cells. In 1/5 cases, CML remission lymphocytes stimulated with autochthonous leukaemic cells showed cytotoxicity to PHA-transformed autochthonous normal lymphoblasts, whilst 1/4 patients showed nonspecific cytotoxicity to L1210 cells when lymphocytes were cultured in MLC or MLTC, as well as in a 3-cell assay.


*Br. J. Cancer* (1981) **43**, 13

### *IN VITRO* GENERATION OF LYMPHOCYTOTOXICITY TO AUTOCHTHONOUS LEUKAEMIC CELLS IN CHRONIC MYELOID LEUKAEMIA

A. G. KHARE, S. H. ADVANI* AND S. G. GANGAL

*From the Immunology Division, Cancer Research Institute and *Tata Memorial Hospital, Tata Memorial Centre, Parel, Bombay* 400012, *India*

Received 14 July 1980 Accepted 10 October 1980

**Summary.—Lymphocytes from 13 chronic myeloid leukaemia (CML) patients in remission were tested for their ability to differentiate *in vitro* into a cell population cytotoxic to autochthonous target leukaemic cells. CML remission lymphocytes were cultured *in vitro* with autochthonous leukaemic cells and allogeneic normal lymphocytes from an unrelated donor, singly or in combination. The cytotoxic lymphocytes obtained after 7 days of culture were tested for their ability to kill autochthonous leukaemic cells in a 3h $^{51}$Cr-release assay. It was found that with the allogeneic stimulus alone, cytotoxicity was generated in 5/13 cases, whilst stimulation of lymphocytes with autochthonous leukaemic cells alone induced cytotoxicity in 7/13 cases. In contrast, anti-leukaemic cytotoxicity was shown in 12/13 cases when responder lymphocytes were stimulated with both autochthonous leukaemic and unrelated allogeneic normal lymphocytes.**

**The specificity of cytotoxicity was confirmed using other targets such as autochthonous PHA-transformed lymphoblasts and mouse L1210 cells. In 1/5 cases, CML remission lymphocytes stimulated with autochthonous leukaemic cells showed cytotoxicity to PHA-transformed autochthonous normal lymphoblasts, whilst 1/4 patients showed nonspecific cytotoxicity to L1210 cells when lymphocytes were cultured in MLC or MLTC, as well as in a 3-cell assay.**

LEUKAEMIA-ASSOCIATED immune reactivity in leukaemic patients has been shown and confirmed by several workers using *in vitro* humoral and cell-mediated immunological parameters (Powles *et al.*, 1971; Leventhal *et al.*, 1972; Harris, 1973; Durantez *et al.*, 1975; Garrett *et al.*, 1977). Among the *in vitro* parameters, lymphocyte-mediated cytotoxicity has often been used for the detection of leukaemia-associated antigens on leukaemic cells (Leventhal *et al.*, 1972; Rosenberg *et al.*, 1972; McCoy *et al.*, 1974). It is, however, difficult to ascertain that the *in vitro* demonstration of target-cell lysis is a function of sensitized T cells. Human leukaemic blasts are possibly unable to induce cytotoxic responses in autologous remission lymphocytes (Zarling *et al.*, 1976; Lee & Oliver, 1978). It was felt that if the stimulator cells did not differ from the responders with respect to LD antigens, proliferative and cytotoxic responses would not be easily generated (Zarling *et al.*, 1976; Zarling & Bach, 1979). Sondel *et al.* (1976) incubated lymphocytes of HLA-identical siblings of patients with acute leukaemia with allogeneic lymphocytes and patients' blasts and demonstrated specific cytotoxicity to leukaemic blasts in 3/4 cases. Zarling *et al.* (1976) also showed that addition of helper stimulus

Correspondence to: Dr (Mrs) Sudha G. Gangal, Officer-in-Charge, Immunology Division, Cancer Research Institute, Tata Memorial Centre, Parel, Bombay 400012, India.

enhances anti-leukaemic cell-mediated cytotoxicity in acute-leukaemic patients. Lee & Oliver (1978) extended this work further to demonstrate *in vitro* generation of cytotoxicity with third-party allogeneic stimulus in 11/14 AML patients. Their experiments confirmed that the cytotoxicity was mediated by T lymphocytes.

Our previous studies on chronic myeloid leukaemia (CML) have shown that CML patients in remission demonstrated cellular sensitization to CML-associated antigens, as tested by *in vitro* lymphocyte blastogenesis and leucocyte migration inhibition assays (Gangal *et al.*, 1976; Gothoskar *et al.*, 1976; Damle *et al.*, 1979). CML remission lymphocytes also showed reactivity to other myeloid or lymphoid leukaemic blast antigens (Gangal *et al.*, 1979). The present investigations were undertaken to find out whether lymphocytes from CML patients in remission are able to differentiate *in vitro* so as to be cytotoxic to autochthonous target leukaemic cells.

### MATERIALS AND METHODS

A total of 13 untreated CML patients were used for this study. The peripheral-blood leucocyte count of these CML patients varied from 15 to $20 \times 10^4$ cells/mm$^3$, the M/E ratio being between 10 and 30 before treatment. The leukaemic cells were cryopreserved at this stage in Dulbecco's medium supplemented with 10% foetal calf serum (FCS, Difco) and 10% dimethyl sulphoxide (DMSO) at a concentration of $1\text{–}2 \times 10^6$ cells/ml in liquid $N_2$. The cryopreserved cells were thawed in two lots, one for *in vitro* sensitization, the second being thawed after 7 days and used for labelling with $^{51}$chromium. The cells were rapidly thawed and slowly suspended and washed in the medium (Dulbecco's medium + 10% FCS + 4 mM/ml L-glutamine). The viability of thawed cells was > 80% in all cases.

In vitro *sensitization.*—The patients attained clinical and haematological remission after busulphan treatment. When they were in complete remission and off the therapy for 1–2 weeks, their peripheral-blood lymphocytes were used for the experiments. Lymphocytes were separated using Ficoll–Hypaque density gradient and washed $\times 3$ in 0·85% saline. The responder cells were finally suspended in culture medium (Dulbecco's medium + 10% FCS + 4 mM glutamine + $5 \times 10^{-5}$M 2-ME and 100 u/ml of penicillin and 50 μg/ml streptomycin) at a concentration of $10^6$ cells/ml. Peripheral-blood lymphocyte suspensions from normal healthy unrelated donors were prepared in a similar way. Autologous thawed leukaemic cells and allogeneic normal lymphocytes were treated with mitomycin-C (MMC, 25 μg for $10^6$ cells) for 45 min, washed and resuspended in culture medium at $10^6$ cells/ml. Responder cells ($4 \times 10^6$) were co-cultured with $4 \times 10^6$ stimulating cells in different combinations, as shown in the Results. When stimulating cells were a mixture of two cell types, each population consisted of $2 \times 10^6$ cells. The cell mixtures consisting of responder and stimulator cells were spun at low speed and incubated at 37°C in a humidified 5% $CO_2$ atmosphere for 6–7 days.

*$^{51}$Cr release assay.*—Frozen autologous leukaemic cells were thawed on the 7th day, washed and resuspended in culture medium. Cells ($2 \times 10^6$) were labelled with 200 μCi of $^{51}$Cr (sodium chromate, sp. act. 15 mCi/mg of sodium chromate, BARC, Bombay, India) for 3 h at 37°C in a water-bath with intermittent shaking. The labelled cells were washed $\times 3$ with tissue-culture medium and suspended in the medium at $10^5$ cells/ml. Other target cells were also labelled with $^{51}$Cr in the same way. Responder cells cultured in different combinations for 7 days were harvested, washed and suspended at a concentration of $10^6$ viable cells/ml in the medium. The viability of responding cell populations varied between 45 and 67%. Viable cells ($10^6$) were mixed with $10^4$ labelled target cells in duplicates. The tubes were spun at a very low speed for 5 min and incubated at 37°C for 3 h. Two tubes were incubated with labelled target cells alone to find out the background (spontaneous) $^{51}$Cr release. Two tubes with $10^4$ target cells were frozen and thawed $\times 5$ in 0·5 ml distilled water to determine the maximum release (100%) of radioactive chromium. After 3 h, 0·3 ml of medium was added to each tube, the cells were resuspended and tubes were centrifuged. Two 0·1ml lots of supernate from each culture was used for counting the release of $^{51}$Cr using a Biogamma Counter (Beckmann).

The percentage cytotoxicity was measured by the standard formula:

$$\% \text{ Cytotoxicity} = \frac{\text{Experimental release} - \text{Mean spontaneous release}}{\text{Mean maximal release} - \text{Mean spontaneous release}} \times 100$$

The spontaneous release of $^{51}Cr$ did not exceed 25% in any of the experiments from which the data are presented and analysed. Percentage cytotoxicity was calculated for each of the quadruplicate samples and expressed as mean % cytotoxicity ± s.e. The data are analysed using Student's *t* test.

### RESULTS

Results of the cytotoxicity assay of cultured lymphocytes from 13 CML patients in remission on autochthonous target leukaemic cells are in Table I. The culture combinations consisted of lymphocytes (a) incubated without stimulator cells, (b) stimulated with allogeneic lymphocytes, (c) incubated with autochthonous leukaemic cells, and (d) incubated with a combination of autochthonous leukaemic cells and allogeneic lymphocytes. All the stimulator cells were treated with MMC. The results are expressed as mean percentage cytotoxicity of quadruplicate samples ± s.e. It can be seen that allogeneic stimulus alone induced remission lymphocytes to express cytotoxicity to autochthonous target leukaemic cells in 5/13 cases, when compared with the cytotoxicity of lymphocytes incubated in culture medium alone, whilst stimulation of lymphocytes with autochthonous leukaemic cells alone induced cytotoxic responses in 7/13 cases. It was interesting to note that in 12/13 cases highly significant cytotoxic responses were obtained when leukaemic cells were used as stimulators along with the third-party allogeneic lymphocyte stimulus. The cytotoxicity in the 3-cell assay was higher than when by stimulating the lymphocytes with autochthonous target cells alone.

In Table II are given the results of experiments on 5 remission lymphocyte samples where PHA-transformed autochthonous lymphoblasts and xenogeneic mouse L1210 cells have also been used as targets, besides autochthonous leukaemic cells. Lymphocyte cultures treated with PHA had 58–65% blasts. It can be seen that in 1/5 tests (AJ 13455) CML remission lymphocytes stimulated with leukaemic cells have shown cytotoxic response towards normal autochthonous PHA-transformed lymphoblasts. In 1/4 tests (AJ 13595) the MLC and MLTC, as

TABLE I.—*Generation of cytotoxicity* in vitro *in CML remission lymphocytes*

| Lymphocyte donors (CML remission patients) | % Cytotoxicity* by cultured lymphocytes stimulated** with | | | |
|---|---|---|---|---|
| | Nil | Allogeneic lymphocytes | Auto CML cells | Allogeneic lymphocytes and auto CML cells |
| AJ 10227 | 10 ± 3 | 30·4 ± 2·1*** | 51·8 ± 3·1*** | 62·2 ± 3·5*** |
| AK 14634 | 15 ± 4 | 34·8 ± 2·5*** | 40·1 ± 2·5*** | 73·9 ± 7·0*** |
| AK 17728 | 8 ± 2 | 48·4 ± 3·7*** | 27·0 ± 3·1*** | 77·2 ± 3·1*** |
| AK 12468 | 26 ± 4 | 32·5 ± 6·0 | 34·2 ± 4·0 | 67·1 ± 4·95*** |
| AH 11282 | 50 ± 6 | 32·2 ± 6·0 | 42·0 ± 5·7 | 48·0 ± 3·2 |
| AJ 930 | 11 ± 2 | 25·2 ± 3·0 | 27·2 ± 1·7*** | 65·1 ± 4·2*** |
| AK 10429 | 20 ± 2 | 18·1 ± 1·6 | 28·5 ± 1·8*** | 52·0 ± 3·1*** |
| AL 2909 | 22 ± 5 | 30·1 ± 1·4 | 40·4 ± 4·5*** | 57·5 ± 4·2*** |
| AH 5593 | 15 ± 3 | 29·9 ± 2·2*** | 32·3 ± 2·3*** | 52·0 ± 3·1*** |
| AH 13595 | 25 ± 4 | 22·0 ± 1·9 | 27·4 ± 2·4 | 39·7 ± 1·4*** |
| AJ 13455 | 25 ± 6 | 28·4 ± 1·6 | 31·6 ± 4·1 | 49·5 ± 1·4*** |
| AK 11024 | 7 ± 2 | 32·8 ± 2·5*** | 36·0 ± 3·3*** | 34·6 ± 1·6*** |
| AL 1021 | 21 ± 5 | 20·1 ± 1·4 | 21·8 ± 1·9 | 36·3 ± 1·9*** |

* Mean ± s.e.
** Stimulator cells treated with MMC.
*** $P < 0·001$ (analysed by Student's *t* test).

2

TABLE II.—*Generation of cytotoxicity* in vitro *in CML remission lymphocytes*

| | | % Cytotoxicity* by cultured lymphocytes stimulated† with | | | |
|---|---|---|---|---|---|
| | Targets | Nil | Allogeneic lymphocytes | Auto CML cells | Allogeneic lymphocytes + Auto CML cells |
| AK 12468§ | Auto CML cells | 26·1 ± 4·0 | 32·5 ± 6·0 | 34·2 ± 4·0 | 67·1 ± 4·9‡ |
| | Auto PHA blasts | 29·0 ± 8·0 | 33·0 ± 4·7 | 31·0 ± 3·8 | 33·9 ± 5·1 |
| | L1210 | 22·0 ± 3·4 | 6·0 ± 1·9 | 6·0 ± 2·9 | 14·0 ± 5·1 |
| AJ 13595 | Auto CML cells | 24·8 ± 4·0 | 22·8 ± 1·9 | 27·4 ± 2·4 | 39·7 ± 1·4‡ |
| | Auto PHA blasts | 30·0 ± 3·0 | 30·0 ± 3·1 | 36·7 ± 6·0 | 32·9 ± 5·9 |
| | L1210 | 6·2 ± 2·1 | 25·0 ± 4·1 ($P < 0·01$) | 26·7 ± 3·8 ($P < 0·002$) | 19·6 ± 3·1 ($P < 0·02$) |
| AH 11282 | Auto CML cells | 50·1 ± 6·0 | 32·2 ± 6·0 | 42·0 ± 5·7 | 48·0 ± 3·2 |
| | Auto PHA blasts | 6·0 ± 1·2 | 8·0 ± 2·0 | 7·0 ± 3·7 | 12·0 ± 3·1 |
| | L1210 | 8·0 ± 2·1 | 2·0 ± 1·1 | 4·0 ± 2·1 | 7·5 ± 1·9 |
| AK 17728 | Auto CML cells | 7·9 ± 2·0 | 48·4 ± 3·7‡ | 27·0 ± 3·1‡ | 77·2 ± 3·1‡ |
| | Auto PHA blasts | 41·1 ± 4·1 | 45·0 ± 8·0 | 43·0 ± 11·0 | 48·1 ± 13·1 |
| | L1210 | N.D. | N.D. | N.D. | N.D. |
| AJ 13455 | Auto CML cells | 25·2 ± 6·0 | 28·4 ± 1·6 | 31·6 ± 4·1 | 49·5 ± 1·4‡ |
| | Auto PHA blasts | 4·5 ± 1·1 | 16·4 ± 5·1 | 22·1 ± 3·1 ($P < 0·002$) | 15·5 ± 4·3 |
| | Auto marrow cells | 18·9 ± 4·4 | 21·4 ± 6·0 | 19·7 ± 3·0 | 39·3 ± 3·0‡ |
| | L1210 | 8·1 ± 2·4 | 6·4 ± 1·8 | 8·7 ± 2·8 | 11·8 ± 3·7 |

* Mean ± s.e. † Stimulator cells tıeated with MMC.
‡ $P < 0·001$ (analysed by Student's $t$ test). § Remission lymphocytes from.

well as 3-cell culture, seemed to induce nonspecific cytotoxicity to mouse L1210 cells. However, the specific leukaemic target cell lysis in these experiments was higher than other targets.

Lymphocytes stimulated *in vitro* were tested on target autochthonous marrow cells in one case (AJ 13455). It is interesting to note that lymphocytes of this patient, when stimulated with autologous leukaemic cells and allogeneic lymphocytes, showed significant cytotoxicity to autologous marrow cells.

### DISCUSSION

In the present series of experiments it has been shown that CML remission lymphocytes can differentiate *in vitro* into a cell population highly cytotoxic to autochthonous leukaemic cells. In our experiments, in 5/13 cases cytotoxicity to autologous target leukaemic cells was displayed by responders stimulated in one-way MLC. Addition of LD stimulus by way of MLC may have caused proliferation and differentiation of cells capable of recognizing target-cell antigens.

Recently, Zarling & Bach (1978) have shown that normal T lymphocytes, sensitized *in vitro* against a pool of allogeneic lymphocytes, lyse autologous EBV-transformed lymphoblastoid cell lines, but not autologous lymphocytes or mitogen-induced blasts. Similarly, they have also demonstrated that peripheral-blood lymphocytes of 2 hairy-cell leukaemic patients, stimulated *in vitro* by a pool of allogeneic lymphocytes from 20 normal donors, kill autologous leukaemia target cells (Zarling *et al.*, 1978). In both these reports, however, it was stressed that lymphocytes from a single allogeneic individual are marginally capable of stimulating T cells to develop into cytotoxic lymphocytes (CTL) cytotoxic to autologous leukaemic or transformed cells, whereas, in our experiments, the proliferation stimulus provided by a single allogeneic cell type appears to be sufficient to generate CTL reactive to autologous leukaemic cells.

In one of their earlier reports, Zarling *et al.* (1976) have demonstrated generation of CTL cytotoxic to autologous leukaemic cells after incubating the lymphocytes with autologous marrow cells and allogeneic lymphocytes. The authors have suggested that the response could be due to the presence of a few leukaemic blasts ($\sim 5\%$) in the marrow. However, it is possible that the response could be due to the stimulus provided by allogeneic cells as shown by us.

Throughout the experiments reported here, lymphocytes have been cultured in medium containing FCS. The possibility that FCS, being mitogenic, may have reactivated the cytotoxic activity in patients' *in vitro* immunized cells cannot be ruled out.

In the group of CML patients investigated by us, a fair number of lymphocytes capable of recognizing target-cell antigens already existed in circulation, since in MLTC-stimulated cultures 8/13 patients responded by displaying specific target-cell lysis. Zarling *et al.* (1976) have shown that when leukaemic blasts are used as stimulators, cytotoxicity was not always demonstrable. Lee & Oliver (1978) have shown that myeloid blasts are poor stimulators even in allogeneic stimulation. The CML leukaemic cell population consists of cells in different stages of maturation, and may express antigens which can be recognized by sensitized lymphocytes. Addition of third-party stimulus has evidently increased the cytotoxicity of CML remission lymphocytes to target leukaemic cells.

In the present investigations, besides autochthonous leukaemic target-cell lysis (shown by 12/13 patients) 1/5 patients showed cytotoxicity to PHA-transformed normal lymphoblasts, and 1/4 patients showed killing of L1210 cells. We have not included stimulator allogeneic normal cells as targets in this study. Zarling *et al.* (1976, 1978) and Lee & Oliver (1978) have shown the lysis of allogeneic stimulator targets by the responders sensitized *in vitro* in a 3-cell assay.

It was interesting to note that lymphocytes of one patient, sensitized *in vitro* in a 3-cell assay, could kill target autochthonous marrow cells. Although this has been demonstrated only in one patient, the findings indicate that the marrow may have retained abnormal cells during remission. Cytogenetic studies on remission marrow cells of CML patients have shown that patients apparently in clinical and haematological remission may still have 20–30% cells with Philadelphia ($Ph^1$) chromosome and other anomalies (Khare *et al.*, unpublished data) indicating the presence of abnormal (leukaemic ?) cells in their marrow.

A number of attempts have recently been made to increase the *in vitro* cytotoxicity of sensitized lymphocytes to specific target cells. These include the use of helper factor produced by primary or secondary MLC (Zarling & Bach, 1979; Wagner, 1978), helper factor produced by mitogen-activated lymphocytes for maintenance of cytotoxic cells *in vitro* (Gills & Smith, 1977) or addition of interferon in the stimulating system (Zarling & Bach, 1979). Using animal models, it has also been possible to prevent tumour growth by mixing tumour cells with CTL generated *in vitro* (Glaser, 1979). Recent evidence suggest that it is possible to maintain specific cytotoxic T cells *in vitro* by repetitive MLC stimulus and mitogen-induced growth factor for 4 months (Gills & Smith, 1977; Zarling & Bach, 1979). The system thus has great potential and applicability in human situations. The present work suggests that in CML it is possible to generate a highly cytotoxic lymphocyte population by using an *in vitro* 3-cell system. Attempts will now be made to maintain the proliferation of CTL specifically reactive to target cells using exogenous growth factors supplied by conditioned medium.

This work was partially funded by the Lady Tata Memorial Trust Research Grant for Leukaemia Research, to whom the authors are grateful for their kind support. We also thank the Chemotherapy Division, Cancer Research Institute, Bombay, for supplying L1210 cells for this study.

### REFERENCES

DAMLE, N. K., KHARE, A. G., ADVANI, S. H. & GANGAL, S. G. (1979) Leukaemia associated *in*

*virro* cell mediated immunity in chronic myeloid leukaemia patients in remission. *Ind. J. Exp. Biol.*, **17**, 1376.

DURANTEZ, A., ZIGHELBOIM, J., THIEME, T. & FAHEY, J. (1975) Antigens shared by leukemic blast cell and lymphoblastoid cell lines detected by lymphocyte dependent antibody. *Cancer Res.*, **35**, 2693.

GANGAL, S. G., DAMLE, N. K., KHARE, A. G. & ADVANI, S. H. (1979) Cellular sensitization in chronic myeloid leukaemia patients to leukemic blast antigens. *Br. J. Cancer*, **40**, 391.

GANGAL, S. G., GOTHOSKAR, B. P., JOSHI, C. S. & ADVANI, S. H. (1976) Demonstration of cellular immunity in chronic myeloid leukemia using leucocyte migration inhibition assay. *Br. J. Cancer*, **33**, 267.

GARRETT, T. J., TAKAHASHI, T. & CLARKSON, B. D. (1977) Detection of antibody to autologous human leukemia cells by immune adherence assays. *Proc. Natl Acad. Sci.*, **74**, 4587.

GILLS, S. & SMITH, K. A. (1977) Long term culture of tumour specific cytotoxic T cells. *Nature*, **268**, 154.

GLASER, M. (1979) Con A mediated *in vitro* activation of lymphocytes primed against syngeneic SV-40 induced tumour associated antigens in mice into secondary effector cells capable of specifically preventing tumour growth. *Cell. Immunol.*, **46**, 201.

GOTHOSKAR, B. P., D'SILVA, H., GHARPURE, H. & ADVANI, S. H. (1976) Leucocyte migration studies in chronic myeloid leukemia (CML). Specificity of reactions. *Eur. J. Cancer*, **12**, 815.

HARRIS, R. (1973) Leukemia antigens and immunity in man. *Nature*, **241**, 95.

LEE, S. K. & OLIVER, R. T. (1978) Autologous leukemia specific T cell mediated lymphocytotoxicity in patients with acute myelogenous leukemia. *J. Exp. Med.*, **147**, 912.

LEVENTHAL, B. G., HALTERMAN, R. H. & HERBERMAN, R. B. (1972) Immune reactivity of leukemia patients to autologous blast cells. *Cancer Res.*, **32**, 1820.

McCOY, L. J., HERBERMAN, R. B., ROSENBERG, E. B., DONNELLY, F. C., LEVINE, P. H. & ALFROD, C. (1974) $^{51}$Chromium release assay foı cell mediated cytotoxicity to human leukemia and lymphoid tissue culture. *Natl Cancer Inst. Monog.*, **37**, 59.

POWLES, R. L., BALCHIN, L. A., FAIRLEY, G. H. & ALEXANDER, P. (1971) Recognition of leukemia cells as foreign befoıe and after autoimmunization. *Br. Med. J.*, i, 486.

ROSENBERG, E. B., HERBERMAN, R. B., LEVINE, P. H., HALTERMAN, R. H., McCOY, J. M. & WUNDERLICH, J. R. (1972) Lymphocyte cytotoxicity reactions to leukemia associated antigens in identical twins. *Int. J. Cancer*, **9**, 648.

SONDELL, P. M., O'BRIEN, C., PORTER, L., SCHLOSSMAN, S. F. & CHESS, L. (1976) Cell mediated destruction of human leukemic cells by MHC identical lymphocytes. *J. Immunol.*, **117**, 2197.

WAGNER, H. (1978) Regulation of Immune response by soluble factors. In *Manipulation of Immune Response in Cancer*. Eds Mitchson & Landy. London: Academic Press. p. 245.

ZARLING, J. M. & BACH, F. H. (1978) Sensitization of lymphocytes against pooled allogeneic cells. *J. Exp. Med.*, **147**, 1334.

ZARLING, J. M. & BACH, F. H. (1979) Continuous cultuıe of T cells cytotoxic for autologous human leukemic cells. *Nature*, **280**, 685.

ZARLING, J. M., RAICH, P. C., McKEOUGH, M. & BACH, F. H. (1976) Generation of cytotoxic lymphocytes "*in vitro*" against autologous human leukemic cells. *Nature*, **262**, 691.

ZARLING, J. M., ROBINS, H. I., RAICH, P. C., BACH, F. H. & BACH, M. L. (1978) Generation of cytotoxic T lymphocytes to autologous human leukemia cells by sensitization to pooled allogeneic normal cells. *Nature*, **247**, 269.